# Phase 2 Study of Pomalidomide (CC-4047) Monotherapy for Children and Young Adults With Recurrent or Progressive Primary Brain Tumors

**DOI:** 10.3389/fonc.2021.660892

**Published:** 2021-06-08

**Authors:** Jason Fangusaro, Maria Giuseppina Cefalo, Maria Luisa Garré, Lynley V. Marshall, Maura Massimino, Bouchra Benettaib, Noha Biserna, Jennifer Poon, Jackie Quan, Erin Conlin, John Lewandowski, Mathew Simcock, Neelum Jeste, Darren R. Hargrave, François Doz, Katherine E. Warren

**Affiliations:** ^1^ Department of Pediatrics, Children’s Healthcare of Atlanta and Aflac Cancer Center at Emory University Medical School, Atlanta, GA, United States; ^2^ Department of Hematology/Oncology and Stem Cell Transplantation, IRCCS Bambino Gesù Children’s Hospital, Rome, Italy; ^3^ Neuro-Oncology Unit, Istituto Giannina Gaslini, Genoa, Italy; ^4^ Children and Young People’s Unit, The Royal Marsden Hospital and The Institute of Cancer Research, London, United Kingdom; ^5^ Pediatric Oncology Unit, Fondazione IRCCS Istituto Nazionale dei Tumori, Milan, Italy; ^6^ Bristol Myers Squibb, Princeton, NJ, United States; ^7^ Celgene Corporation, Uxbridge, United Kingdom; ^8^ Pediatric Oncology Unit, UCL Great Ormond Street Hospital for Children, London, United Kingdom; ^9^ Department of Pediatric Oncology, Institut Curie and University of Paris, Paris, France; ^10^ National Cancer Institute, National Institutes of Health, Bethesda, MD, United States

**Keywords:** diffuse intrinsic pontine glioma, ependymoma, high-grade glioma, medulloblastoma, pomalidomide, progressive or recurrent disease

## Abstract

**Introduction:**

Treatment of recurrent primary pediatric brain tumors remains a major challenge, with most children succumbing to their disease. We conducted a prospective phase 2 study investigating the safety and efficacy of pomalidomide (POM) in children and young adults with recurrent and progressive primary brain tumors.

**Methods:**

Patients with recurrent and progressive high-grade glioma (HGG), diffuse intrinsic pontine glioma (DIPG), ependymoma, or medulloblastoma received POM 2.6 mg/m^2^/day (the recommended phase 2 dose [RP2D]) on days 1-21 of a 28-day cycle. A Simon’s Optimal 2-stage design was used to determine efficacy. Primary endpoints included objective response (OR) and long-term stable disease (LTSD) rates. Secondary endpoints included duration of response, progression-free survival (PFS), overall survival (OS), and safety.

**Results:**

46 patients were evaluable for response (HGG, n = 19; DIPG, ependymoma, and medulloblastoma, n = 9 each). Two patients with HGG achieved OR or LTSD (10.5% [95% CI, 1.3%-33.1%]; 1 partial response and 1 LTSD) and 1 patient with ependymoma had LTSD (11.1% [95% CI, 0.3%-48.2%]). There were no ORs or LTSD in the DIPG or medulloblastoma cohorts. The median PFS for patients with HGG, DIPG, ependymoma, and medulloblastoma was 7.86, 11.29, 8.43, and 8.43 weeks, respectively. Median OS was 5.06, 3.78, 12.02, and 11.60 months, respectively. Neutropenia was the most common grade 3/4 adverse event.

**Conclusions:**

Treatment with POM monotherapy did not meet the primary measure of success in any cohort. Future studies are needed to evaluate if POM would show efficacy in tumors with specific molecular signatures or in combination with other anticancer agents.

**Clinical Trial Registration:**

ClinicalTrials.gov, identifier NCT03257631; EudraCT, identifier 2016-002903-25.

## Introduction

Central nervous system (CNS) tumors represent the second most common pediatric cancer and remain the leading cause of childhood cancer-related mortality (1–3). In children and adolescents, high-grade glioma (HGG World Health Organization grades III and IV), diffuse intrinsic pontine glioma (DIPG), medulloblastoma, and ependymoma represent the majority of malignant primary brain and CNS tumors (4–6). The 5-year overall survival (OS) rate for patients with HGG ranges between 10% to 20% while the OS rate for DIPG is less than 10% to 15% (7). In addition, most patients with recurrent medulloblastoma and ependymoma will die from progressive disease despite treatment (5, 6). The need for alternative and efficacious treatment options is further compounded by treatment-associated morbidities with treatments such as radiation and classic cytotoxic chemotherapies, which can impact a child’s quality of life and functional outcomes (8–14).

Novel agents with unique mechanisms of action may help to overcome these barriers. Immunomodulatory agents, including pomalidomide (POM), thalidomide, and lenalidomide, have demonstrated anti-inflammatory properties (including T-cell activation and proinflammatory cytokine inhibition), angiogenesis inhibition, and induction of antiproliferative activities (15–23). Furthermore, POM has been shown to penetrate the blood-brain barrier (24). The multimodal mechanism of action and ability to cross the blood-brain barrier suggest that POM may represent a unique approach for addressing the unmet needs in primary pediatric CNS tumors.

A Pediatric Brain Tumor Consortium (PBTC) phase 1 trial of pediatric patients with recurrent, refractory, or progressive primary CNS tumors demonstrated tolerability of lenalidomide at doses exceeding those in adults as well as evidence of activity within the confines of a phase 1 study (25). Myelosuppression was the most common adverse event (AE) during the dose-finding part of the study (25). Another PBTC phase 1 study in children with recurrent, progressive/refractory CNS tumors identified the POM maximum-tolerated dose as 2.6 mg/m^2^; diarrhea, thrombocytopenia, and lung infection were dose-limiting toxicities (26). Subsequently, 12 additional patients were enrolled based on age and steroid use, and there was no obvious difference in tolerability observed based on these factors (26). POM exposure increased in a dose-dependent manner, similar to what has been observed in adults (26). In this trial, one patient with an oligodendroglioma achieved long-term stable disease (LTSD) and one patient with an anaplastic pleomorphic xanthoastrocytoma achieved a partial response (PR).

The preliminary safety and efficacy data in this PBTC phase 1 study led to the development of the current phase 2 study where we investigated safety and efficacy of POM in children and young adults with recurrent or progressive primary CNS tumors at the RP2D.

## Materials and Methods

### Study Oversight

The study was approved by the institutional review board or ethics committee at each participating study site prior to initiation. This study was conducted in accordance with the Declaration of Helsinki and Good Clinical Practice Guidelines of the International Council for Harmonisation. Written informed consent (and assent when appropriate) was obtained from each patient and/or their legal guardian prior to study entry. The protocol is included in the **Supplementary Materials**.

### Patients

Eligible patients included those aged 1 to < 21 years with a diagnosis of recurrent or progressive primary HGG, DIPG, ependymoma, or medulloblastoma. Patients must have received ≥1 prior standard therapy (or a generally accepted upfront therapy if no standard existed) and have no known curative therapeutic alternative. Other key inclusion criteria were tumor located in the brain, histologic verification at the time of either diagnosis or recurrence (patients with DIPG were exempt from histologic verification if they had typical clinical course and magnetic resonance imaging [MRI] findings of DIPG), and measurable disease (primary brain tumor that was measurable in 2 perpendicular diameters on MRI). Patients were required to have a Lansky or Karnofsky functional performance status score ≥ 50 at screening, as well as adequate renal, hepatic, pulmonary, and bone marrow function. Prior to enrollment, patients must have recovered from any clinically significant acute treatment-related AEs associated with prior therapies and had no significant worsening in clinical status for a minimum of 7 days prior to the first dose of POM.

### Treatment

Patients started POM at the RP2D of 2.6 mg/m^2^/day once daily on days 1-21 of each 28-day treatment cycle, followed by a 7-day rest period (26). Treatment could continue for up to 24 cycles or until progressive disease, consent withdrawal, treatment intolerance, or death.

### Study Design and Power Calculation

This phase 2, multicenter, international, open-label, parallel-group study assessed POM using a Simon’s Optimal 2-stage design (**Supplementary Figure 1**). Under Simon’s Optimal 2-stage design with a 5% significance level and 90% power, assuming a lower boundary of interest in the objective response (OR) and long-term stable disease rate of 10% and an upper boundary of interest in the OR and LTSD rate of 40%, a total of 20 patients evaluable for the primary endpoint were required per cohort: 9 in stage 1 and an additional 11 in stage 2.

In stage 1, 9 patients were enrolled for each primary brain tumor type (cohort). During stage 1, if ≥ 2 patients in any given cohort achieved either an OR (complete response or PR) within the first 6 cycles of treatment (first 3 cycles for DIPG) or achieved LTSD (maintained for ≥ 6 cycles [≥ 3 cycles for DIPG]), an additional 11 patients were enrolled for a total of 20 patients per cohort. During stages 1 and 2, if ≥ 5 patients among the 20 in a given cohort achieved either OR or LTSD within the specified time, POM would be considered effective in that disease indication. The study was registered at ClinicalTrials.gov (NCT03257631) and EudraCT (2016–002903–25).

### Endpoints

The primary endpoint was the proportion of patients achieving either OR or LTSD. The secondary endpoints were duration of response (DOR), progression-free survival (PFS), and OS (all of which were assessed using Kaplan-Meier curves) as well as safety. POM pharmacokinetics was an exploratory endpoint. Efficacy endpoints were assessed in the response population, which included all enrolled patients who received ≥ 1 cycle of POM or who withdrew prior to completing 1 cycle of POM due to disease progression; patients who withdrew treatment for any reason other than disease progression prior to completing 1 cycle of POM were replaced. Treatment exposure, dose modification, and safety data were assessed in the safety population.

### Assessments and Follow-Up

Brain tumor assessments were conducted by standard MRI (with and without contrast) using 3 sequences (T1-weighted pre- and postcontrast, T2-weighted, and fluid-attenuated inversion recovery). Brain MRI assessments were conducted during screening and then on day 1 of cycles 3, 5, 7, 10, 13, 16, 19, and 22; after completion of cycle 24; and as clinically indicated. For patients with DIPG only, post-baseline brain MRI assessments were performed on day 1 of cycles 4, 7, 10, 13, 16, 19, and 22; after completion of cycle 24; and as clinically indicated. Radiographic OR was assessed using the sequence best representative of the tumor in the opinion of the neuroradiologist (the same sequence was used for serial measurements). Patients who did not meet the criteria for response or disease progression by the end of cycle 6 (end of cycle 3 for DIPG) were considered to have LTSD.

Response evaluations were assessed both locally and by an independent central reviewer; the local investigator assessment was used for patient eligibility and treatment decisions. Efficacy-based endpoints incorporating tumor assessments were based on the independent central assessment. For DOR, PFS, and OS, median values and corresponding 95% CIs were estimated using Kaplan-Meier methods.

Adverse events were coded according to the Medical Dictionary for Regulatory Activities. The severity and intensity of AEs were graded based upon patient symptoms according to the National Cancer Institute Common Terminology Criteria for AEs (version 4.03). Laboratory assessments were performed locally and at each scheduled visit.

Whole blood samples were collected for pharmacokinetics analyses at the time of POM administration (pre-dose) and 2 hours following POM administration on days 8 and 15 of cycle 1. Plasma concentrations of POM were summarized by geometric mean and geometric coefficient of variation.

After POM discontinuation, patients were followed every 3 months (± 14 days) from the 28-day post-treatment safety follow-up visit for second primary malignancies (regardless of causal relationship), any drug-related serious AEs, OS, and start of any new anticancer therapies. Follow-up continued for up to 5 years after last patient enrollment unless a patient withdrew consent, was lost to follow-up, or died.

## Results

### Patient Disposition and Baseline Characteristics

**Figure 1:**
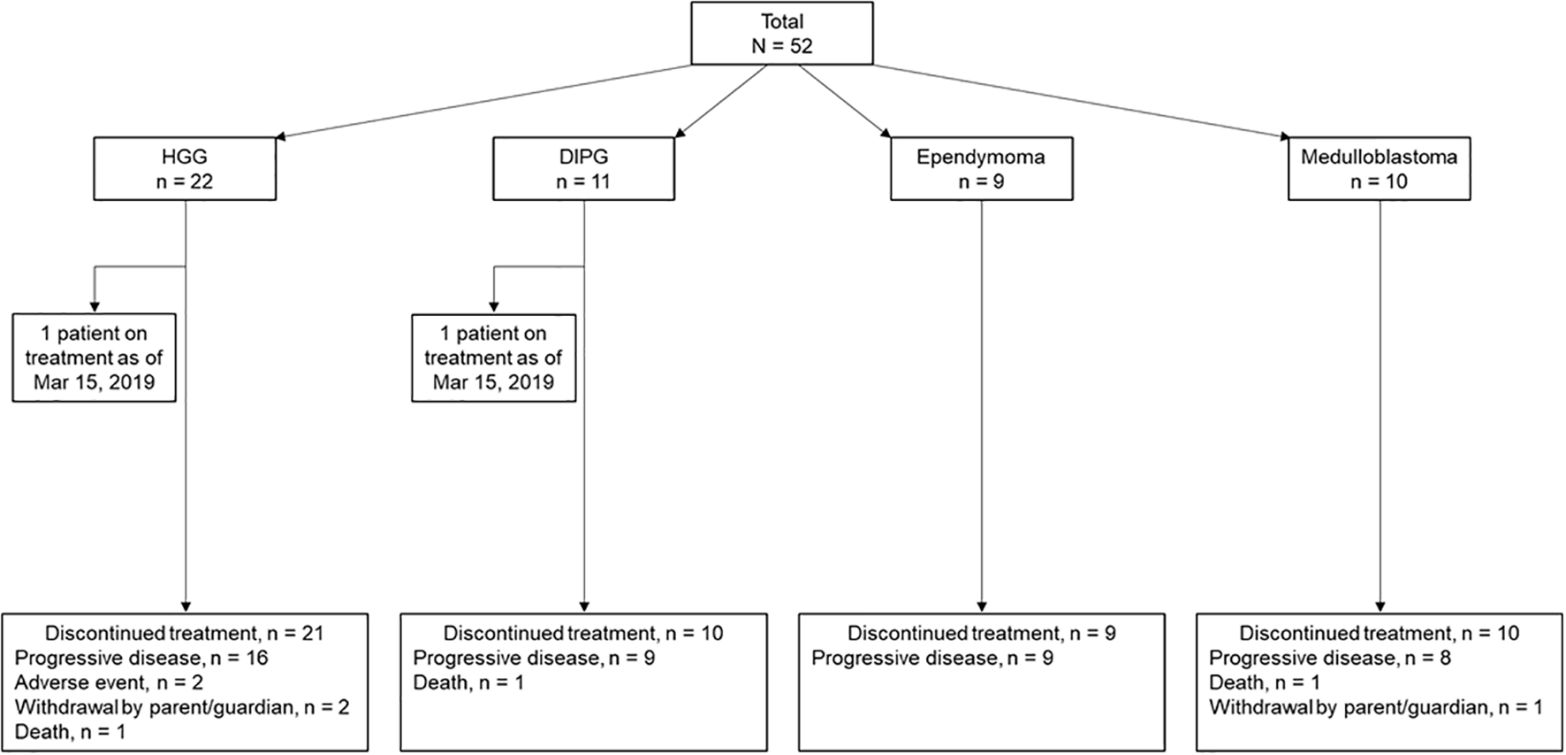
Patient Disposition by Tumor Type (safety population).

Of 57 patients who were screened for eligibility, 53 were enrolled at 18 institutions in France (n = 5), Italy (n = 3), Spain (n = 2), the United Kingdom (n = 3), and the United States (n = 5). Four patients were screened but not enrolled due to screen failure (n = 3) and death (n = 1). One enrolled patient did not receive study treatment. Patients were treated between August 2017 and March 2019. As of the database cutoff date (March 15, 2019), 2 patients were still on treatment (1 each in the HGG and DIPG cohorts [the patient with DIPG was not part of the response population]). The remaining 50 patients discontinued POM treatment due to progressive disease (84.0%), death (6.0%), withdrawal by parent or guardian (6.0%), or AE (4.0%) (**Figure 1**). The response population consisted of 46 patients (19 patients with HGG; 9 patients each with DIPG, ependymoma, or medulloblastoma).

Baseline characteristics are reported in **Table 1**. The median age was 11.5 years (range, 4-18 years), and most patients were male (63.5%). Overall, patients received a median of 3 (range, 1-17) previous systemic therapies.

**Table 1:** Demographics and Baseline Characteristics (safety population).

Characteristic	HGG n = 22	DIPG n = 11	Ependymoma n = 9	Medulloblastoma n = 10	Total n = 52
**Age, median (range), years**	13.5 (5-18)	7.0 (4-12)	12.0 (4-15)	10.0 (4-17)	11.5 (4-18)
≥ 1 to < 6, n (%)	1 (4.5)	1 (9.1)	2 (22.2)	1 (10.0)	5 (9.6)
≥ 6 to < 12, n (%)	5 (22.7)	9 (81.8)	1 (11.1)	6 (60.0)	21 (40.4)
≥ 12, n (%)	16 (72.7)	1 (9.1)	6 (66.7)	3 (30.0)	26 (50.0)
**Sex, n (%)**					
Male	14 (63.6)	7 (63.6)	5 (55.6)	7 (70.0)	33 (63.5)
Female	8 (36.4)	4 (36.4)	4 (44.4)	3 (30.0)	19 (36.5)
**Prior lines of therapy**					
Radiation therapy					
n	22	11	9	10	52
Median (range)	1.0 (1-2)	1.0 (1-3)	2.0 (1-4)	1.0 (1-3)	1.0 (1-4)
Surgery					
n	21	5	9	10	45
Median (range)	1.0 (1-4)	1.0 (1-2)	3.0 (1-4)	2.0 (1-5)	2.0 (1-5)
Systemic therapy^a^					
n	20	7	8	10	45
Median (range)	2.0 (1-7)	2.0 (1-4)	4.0 (2-7)	7.0 (3-17)	3.0 (1-17)
Stem cell transplants (autologous)					
n	0	0	0	3	3
Median (range)	NA	NA	NA	1.0 (1-4)	1.0 (1-4)
Lansky performance status score^a^					
n	15	11	9	9	44
Median (range)	80.0 (60-100)	80.0 (50-100)	100.0 (70-100)	90.0 (70-100)	90.0 (50-100)
90-100, n (%)	7 (31.8)	3 (27.3)	7 (77.8)	7 (70.0)	24 (46.2)
70-80, n (%)	5 (22.7)	6 (54.5)	2 (22.2)	2 (20.0)	15 (28.8)
50-60, n (%)	3 (13.6)	2 (18.2)	0	0	5 (9.6)
Karnofsky performance status score^b^					
n	7	0	0	1	8
Median (range)	80.0 (60-100)	NA	NA	90.0 (90-90)	85.0 (60-100)
90-100, n (%)	3 (13.6)	NA	NA	1 (10.0)	4 (7.7)
70-80, n (%)	2 (9.1)	NA	NA	0	2 (3.8)
50-60, n (%)	2 (9.1)	NA	NA	0	2 (3.8)

DIPG, diffuse intrinsic pontine glioma; HGG, high-grade glioma; NA, not applicable.

a Includes anti-epidermal growth factor receptor monoclonal antibody (mAb)/inhibitors, chemotherapy, anti-vascular endothelial growth factor mAbs, mammalian target of rapamycin inhibitors, immunomodulatory agents, immune checkpoint inhibitors, and/or B-Raf inhibitors.

b Lansky performance status score was collected for patients < 16 years of age; Karnofsky performance status score was collected for patients ≥ 16 years of age.

### Efficacy

The median follow-up time for all patients was 4.86 months (range, 0.6-17.2 months). For the primary analyses (response population), the OR and LTSD rates were 10.5% (1 PR and 1 LTSD) for HGG and 11.1% (1 LTSD) for ependymoma (**Table 2**). All 3 patients with PR or LTSD had received radiation treatment as part of an upfront therapy. No OR or LTSD was recorded in the DIPG or medulloblastoma cohorts.

**Table 2:** Objective Response and Long-Term Stable Disease per Independent Central Review (response population).

Parameter	HGG n = 19	DIPG n = 9	Ependymoma n = 9	Medulloblastoma n = 9
Rate of objective response or long-term stable disease^a^				
n (%)	2 (10.5)	0	1 (11.1)	0
95% CI	(1.3-33.1)	(0.0-33.6)	(0.3-48.2)	(0.0-33.6)
**Objective response, n (%)**	1 (5.3)	0	0	0
Long-term stable disease, n (%)^a^	1 (5.3)	0	1 (11.1)	0
**Best overall response, n (%)**				
Complete response	0	0	0	0
Partial response	1 (5.3)	0	0	0
Stable disease	1 (5.3)	0	3 (33.3)	1 (11.1)
≥ 3 cycles	1 (5.3)	0	3 (33.3)	1 (11.1)
≥ 6 cycles	1 (5.3)	0	1 (11.1)	0
Disease progression	11 (57.9)	6 (66.7)	6 (66.7)	5 (55.6)
Not evaluable^b^	6 (31.6)	3 (33.3)	0	3 (33.3)

DIPG, diffuse intrinsic pontine glioma; HGG, high-grade glioma.

a Long-term stable disease was defined as stable disease maintained for ≥ 6 cycles (≥ 3 cycles for DIPG).

b Patients who discontinued study treatment due to disease progression or relapse prior to disease assessment were considered not evaluable with regard to the primary endpoint.

The independently assessed PFS analysis was based on 17 (89.5%), 9 (100.0%), 9 (100.0%), and 8 (88.9%) events for patients in the response population with HGG, DIPG, ependymoma, and medulloblastoma, respectively (**Table 3**). The median PFS values were 7.86, 11.29, 8.43, and 8.43 weeks, respectively. The OS analysis was based on 12 (63.2%), 7 (77.8%), 5 (55.6%), and 4 (44.4%) events for patients in the response population with HGG, DIPG, ependymoma, and medulloblastoma, respectively (**Table 3**); the median OS values were 5.06, 3.78, 12.02, and 11.60 months, respectively.

**Table 3:** Progression-Free Survival and Overall Survival (response population).

	HGG (n = 19)	DIPG (n = 9)	Ependymoma (n = 9)	Medulloblastoma (n = 9)
**Progression-free survival per independent central review**				
Events, n (%)	17 (89.5)	9 (100.0)	9 (100.0)	8 (88.9)
Time to event, median (95% CI), weeks^a^	7.86 (5.14-8.14)	11.29 (2.86-12.57)	8.43 (5.57-16.14)	8.43 (7.29-18.00)
Event-free rate, % (SE)				
Week 4	78.9 (9.35)	88.9 (10.48)	100.0	100.0
Week 8	34.0 (11.20)	66.7 (15.71)	66.7 (15.71)	66.7 (15.71)
Week 16	11.3 (7.54)	0	33.3 (15.71)	27.8 (16.17)
Week 24	11.3 (7.54)	─	11.1 (10.48)	0
Week 32	5.7 (5.50)	─	0	─
**Overall survival**				
Events, n (%)	12 (63.2)	7 (77.8)	5 (55.6)	4 (44.4)
Time to event, median (95% CI), months^a^	5.06 (2.04-11.63)	3.78 (0.66-NA)	12.02 (2.86-NA)	11.60 (1.74-NA)
Event-free rate, % (SE)				
Month 3	65.7 (11.50)	55.6 (16.56)	88.9 (10.48)	88.9 (10.48)
Month 6	44.3 (12.81)	33.3 (15.71)	59.3 (18.48)	63.5 (16.92)
Month 9	35.5 (12.96)	22.2 (13.86)	59.3 (18.48)	63.5 (16.92)
Month 12	11.8 (10.58)	22.2 (13.86)	59.3 (18.48)	42.3 (20.64)

DIPG, diffuse intrinsic pontine glioma; HGG, high-grade glioma; NA, not available; SE, standard error.

a Median time to event based on Kaplan-Meier product-limit estimates.

### Treatment Exposure and Dose Modifications

The median treatment durations for patients in the safety population with HGG, DIPG, ependymoma, and medulloblastoma were 40.5 (range, 11-532), 84.0 (range, 7-448), 112.0 (range, 28-252), and 57.0 (range, 28-118) days, respectively. Patients received a median of 2.0 (range, 1-19), 3.0 (range, 1-16), 4.0 (range, 1-9), and 2.0 (range, 1-4) treatment cycles, respectively. Cumulative treatment exposure and dose intensity data are reported in **Supplementary Table 1**.

Four patients had dose reductions (HGG, n = 2; ependymoma, n = 1; and medulloblastoma, n = 1). One patient with HGG required a dose reduction for AEs (febrile neutropenia, pneumonia, and neutropenia). Six patients had dose interruptions (HGG, n = 1; ependymoma, n = 3; and medulloblastoma, n = 2). AEs were the primary reason for dose interruptions (4 of 6 patients; 1 patient each had a dose interruption due to forgetfulness [ependymoma] and forgot/missed dose [medulloblastoma]). The AEs leading to dose interruption were diarrhea and hydrocephalus (2 patients each), anemia, neutropenia, thrombocytopenia, and vomiting (1 patient each).

### Safety

Overall, 63.6%, 45.5%, 77.8%, and 80.0% of patients with HGG, DIPG, ependymoma, and medulloblastoma, respectively, experienced a treatment-emergent AE (TEAE) related to POM (**Table 4**); the corresponding rates for grade 3/4 TEAEs related to POM were 45.5%, 27.3%, 22.2%, and 40.0%. The most common grade 3/4 TEAE related to POM was neutropenia. The rates of grade 3/4 neutropenia for patients with HGG, DIPG, ependymoma, and medulloblastoma were similar across disease cohorts: 31.8%, 27.3%, 22.2%, and 30.0%, respectively. Other frequent TEAEs related to POM are summarized in **Table 4**. Overall, 6 patients (27.3%) with HGG and 1 (9.1%) with DIPG experienced ≥ 1 serious TEAE related to POM.

**Table 4:** Safety (safety population).

Safety parameter, n (%)	HGG n = 22	DIPG n = 11	Ependymoma n = 9	Medulloblastoma n = 10
Patients with ≥ 1 TEAE related to POM	14 (63.6)	5 (45.5)	7 (77.8)	8 (80.0)
Patients with ≥ 1 grade 3/4 TEAE related to POM	10 (45.5)	3 (27.3)	2 (22.2)	4 (40.0)
Patients with ≥ 1 serious TEAE related to POM	6 (27.3)	1 (9.1)	0	0
TEAEs (any grade) related to POM^a^				
Neutropenia	9 (40.9)	3 (27.3)	7 (77.8)	4 (40.0)
Leukopenia	6 (27.3)	3 (27.3)	6 (66.7)	3 (30.0)
Lymphopenia	6 (27.3)	1 (9.1)	5 (55.6)	0
Thrombocytopenia	6 (27.3)	0	3 (33.3)	3 (30.0)
Anemia	5 (22.7)	0	3 (33.3)	2 (20.0)
Alanine aminotransferase level increased	2 (9.1)	1 (9.1)	1 (11.1)	2 (20.0)
Constipation	3 (13.6)	0	1 (11.1)	2 (20.0)
Maculopapular rash	1 (4.5)	0	3 (33.3)	2 (20.0)
Pruritus	0	2 (18.2)	2 (22.2)	1 (10.0)
Fatigue	1 (4.5)	0	1 (11.1)	2 (20.0)
Decreased appetite	0	1 (9.1)	0	2 (20.0)
Dry skin	0	1 (9.1)	2 (22.2)	0
Vomiting	1 (4.5)	0	0	2 (20.0)
Grade 3/4 TEAEs related to POM^b^				
Neutropenia	7 (31.8)	3 (27.3)	2 (22.2)	3 (30.0)
Lymphopenia	2 (9.1)	1 (9.1)	0	0
Febrile neutropenia	2 (9.1)	0	0	0
Leukopenia	1 (4.5)	1 (9.1)	0	0
Thrombocytopenia	2 (9.1)	0	0	0
Hypokalemia	0	0	0	1 (10.0)
Vertigo	0	1 (9.1)	0	0

DIPG, diffuse intrinsic pontine glioma; HGG, high-grade glioma; POM, pomalidomide; TEAE, treatment-emergent adverse event.

a ≥ 20% incidence for any tumor type.

b ≥ 5% incidence for any tumor type.

Ten patients from the safety population died during the treatment period of the study; 9 of those deaths were due to progressive disease (HGG, n = 5; DIPG, n = 2; ependymoma, n = 1; medulloblastoma, n = 1), and 1 was due to an AE (sepsis; patient with DIPG). The investigator concluded the sepsis (grade 4 and subsequent death) was not treatment-related. During follow-up, 20 additional patients from the safety population died due to progressive disease (HGG, n = 7; DIPG, n = 5; ependymoma, n = 4; medulloblastoma, n = 4).

### Pharmacokinetics

Plasma concentrations of POM by tumor type are reported in **Supplementary Table 2**. No clear differences in POM exposure were observed between the different tumor types.

## Discussion

The current study did not demonstrate the necessary level of clinically meaningful activity of POM monotherapy based on the original statistical design in children and young adults with recurrent or progressive HGG, DIPG, ependymoma, and medulloblastoma. The HGG cohort met the protocol-defined criteria for advancement to stage 2; however, this was the only cohort to advance to stage 2, and the criteria for reaching a threshold of efficacy interest for POM in the Simon stage 2 were not met. The safety profile of POM was generally consistent with previous findings in adults and children (26), with neutropenia being the most common grade 3/4 TEAE related to POM.

In the current study, 1 patient with progressive HGG at study entry achieved a PR and received treatment for > 1.5 years, and 2 patients (1 with HGG, 1 with ependymoma) experienced LTSD. Despite the overall discouraging findings with POM monotherapy, these data suggest some activity potentially worth further investigation. Identifying patients who could potentially benefit from combining POM with other anticancer agents may enhance the level of activity observed in clinical trials. A phase 1 trial examined the combination of dasatinib, lenalidomide, and temozolomide in pediatric patients with either relapsed or refractory CNS tumors (27). The trial established feasibility of the combination; however, any efficacy data were preliminary, and it remains to be determined whether an efficacy benefit exists. It is also unclear if specific tumor molecular signatures may be more responsive to POM as this was not explored in the current study. The inclusion of molecular testing in ongoing clinical trials may lead to the identification of potential driver mutations of pediatric CNS tumors that can inform therapeutic decisions (28). For example, our current understanding of HGG tumors is that they can be categorized into 4 epigenetic subgroups (29–31), including the common histone 3 K27M mutation that disrupts H3K27 methylation and acetylation, causing widespread gene dysregulation (32). The combination of POM and histone deacetylase inhibitors or H3K27 methyltransferase inhibitors demonstrated antitumor activity in preclinical models of multiple myeloma (33, 34) and could be considered in pediatric CNS tumors.

Beyond immunomodulatory therapies, additional efforts are ongoing to investigate immune checkpoint inhibitor-based regimens and targeted therapies. For example, the combination of checkpoint inhibitors with low-dose radiotherapy or chemotherapy (e.g., NCT03585465, NCT03690869, NCT02989636) or other types of immunotherapy, such as chimeric antigen receptor T cells and cancer vaccines (e.g., NCT03500991, NCT03638167, NCT04185038 NCT04239040) are being investigated in pediatric patients (34). Additionally, targeted therapies (i.e., BRAF, MEK and TRK inhibitors) have demonstrated promising activity in pediatric brain tumors (35, 36).

The safety profile of POM in the current study was generally consistent with previous findings in adults and children. The grade 3/4 TEAEs related to POM were mainly hematologic in nature, and the most common was neutropenia. Interestingly, the medulloblastoma cohort, a patient population typically treated with craniospinal radiotherapy, had a similar incidence of myelosuppression as that of the cohorts not typically treated with craniospinal radiotherapy. Previously published studies of POM and lenalidomide in pediatric patients with recurrent, refractory, or progressive CNS tumors also reported hematologic AEs (25, 26).

This study is limited by the relatively small sample size; however, the tolerability, safety, and failure to achieve threshold antitumor activity in this setting are generally consistent with previous findings in patients with recurrent or progressive CNS tumors. The lack of clinically meaningful efficacy in this patient population underscores the urgent need for efficacious treatments and a better understanding of the specific antitumor mechanisms of POM. Future efforts should focus on understanding tumor molecular profiles and combination therapy with other cytotoxic, molecular, and immunomodulatory compounds.

## Data Availability Statement

Bristol Myers Squibb policy on data sharing may be found at https://www.bms.com/researchers-and-partners/independent-research/data-sharing-request-process.html.

## Ethics Statement

The study was approved by the institutional review board or ethics committee at each participating study site prior to initiation. This study was conducted in accordance with the Declaration of Helsinki and Good Clinical Practice Guidelines of the International Council for Harmonisation. Written informed consent (and assent when appropriate) was obtained from each patient and/or their legal guardian prior to study entry. The protocol is included in the **Supplementary Materials**.

## Author Contributions

Conception or design of the work [JF, FD, KEW, MS, JL, NB, and BB] or the acquisition, analysis, or interpretation of data for the work [all authors]. Drafting the manuscript or revising it critically for important intellectual content [all authors]. Agreement to be accountable for all aspects of the work in ensuring that questions related to the accuracy or integrity of any part of the work are appropriately investigated and resolved [all authors]. All authors contributed to the article and approved the submitted version.

## Funding

This study was funded by Bristol Myers Squibb.

## Conflict of Interest

BB, NB, JP, JQ, EC, JL and NJ were employed by Bristol Myers Squibb. MS is employed by and has stock ownership with Celegne. JF: Related to the work: Celgene (a Bristol-Myers Squibb Company) (advisory board). LM: Honoraria/consulting/advisory boards for Bristol-Myers Squibb, Bayer, Eisai, Tesaro. MM: Bristol-Myers Squibb (advisory boards and travel expenses), Roche (advisory board), Novartis (advisory board), Oncoscience (advisory board). BB, NB, JP, JQ, EC, JL and NJ: Employment and stock ownership with Bristol-Myers Squibb Company. DH: Related to the work: Celgene (a Bristol-Myers Squibb Company) (advisory board). Not related to the work: Consulting, AstraZeneca, Bayer, Boehringer Ingelheim, Novartis, Roche/Genentech; research funding, AstraZeneca; expert testimony, AstraZeneca; travel, Boehringer Ingelheim, Novartis, Roche/Genentech; other, AbbVie, Bristol-Myers Squibb, and Novartis. FD: Related to the work: Celgene (a Bristol-Myers Squibb Company) (advisory board). Not related to the work: Bayer (advisory boards and travel expenses), Bristol-Myers Squibb (advisory boards and travel expenses), Roche (advisory board and travel expenses), Loxo Oncology (advisory board), Novartis (advisory board), Tesaro (advisory board), Servier (advisory boards and consulting). KW: Related to the work: Celgene (a Bristol-Myers Squibb Company) (advisory board). Not related to the work: Celgene (a Bristol-Myers Squibb Company) (clinical trial sponsorship).

The remaining authors declare that the research was conducted in the absence of any commercial or financial relationships that could be construed as a potential conflict of interest.

The authors declare that this study received funding from Bristol Myers Squibb. The sponsor was involved in the study design, collection, analysis, interpretation of data, and funded the writing of this article.
