# Supplementary material for: Phase 2 Study of Pomalidomide (CC-4047) Monotherapy for Children and Young Adults With Recurrent or Progressive Primary Brain Tumors
Source: Front Oncol. 2021 Jun 8;11:660892. doi: 10.3389/fonc.2021.660892 (PMC8218626; doi:10.3389/fonc.2021.660892)
Supplement: Supplementary file 1 [file DataSheet_1.pdf]

Supplemental Materials

Supplementary Table 1 Cumulative Exposure and Dose Intensity (safety population)

Parameter, median (range)	HGG n = 22	DIPG n = 11	Ependymoma n = 9	Medulloblastoma n = 10
Cumulative dose, mg/m^2	67.26 (29.8-1011.4)	157.15 (18.9-860.9)	205.53 (55.6-479.4)	112.81 (56.8-208.8)
Relative dose intensity[a]	1.01 (0.6-1.4)	0.99 (0.8-1.4)	1.02 (0.9-1.1)	0.98 (0.9-1.1)

DIPG, diffuse intrinsic pontine glioma; HGG, high-grade glioma.

[a] Defined as dose intensity divided by planned dose intensity.

Supplementary Table 2 Summary of POM Plasma Concentrations by Disease Indication and Scheduled Time Point (pharmacokinetics population)

Geometric mean (geometric CV%)	HGG		DIPG		Ependymoma		Medulloblastoma	
	Cycle 1 Day 8 (n = 14)	Cycle 1 Day 15 (n = 14)	Cycle 1 Day 8 (n = 10)	Cycle 1 Day 15 (n = 9)	Cycle 1 Day 8 (n = 6)	Cycle 1 Day 15 (n = 8)	Cycle 1 Day 8 (n = 8)	Cycle 1 Day 15 (n = 8)
Predose	21.48 (95.0)	14.72 (103.2)	11.24 (220.0)	10.67 (273.2)	22.23 (105.5)	8.72 (323.7)	9.0 (144.5)	10.38 (94.3)
2 hours postdose	66.40 (119.2)	60.80 (61.3)	61.10 (108.6)	62.04 (106.7)	77.20 (90.0)	97.88 (9.4)	89.71 (64.1)	77.08 (61.9)

CV, coefficient of variation; DIPG, diffuse intrinsic pontine glioma; HGG, high-grade glioma; POM, pomalidomide.

Supplementary Fig. 1 Study Design

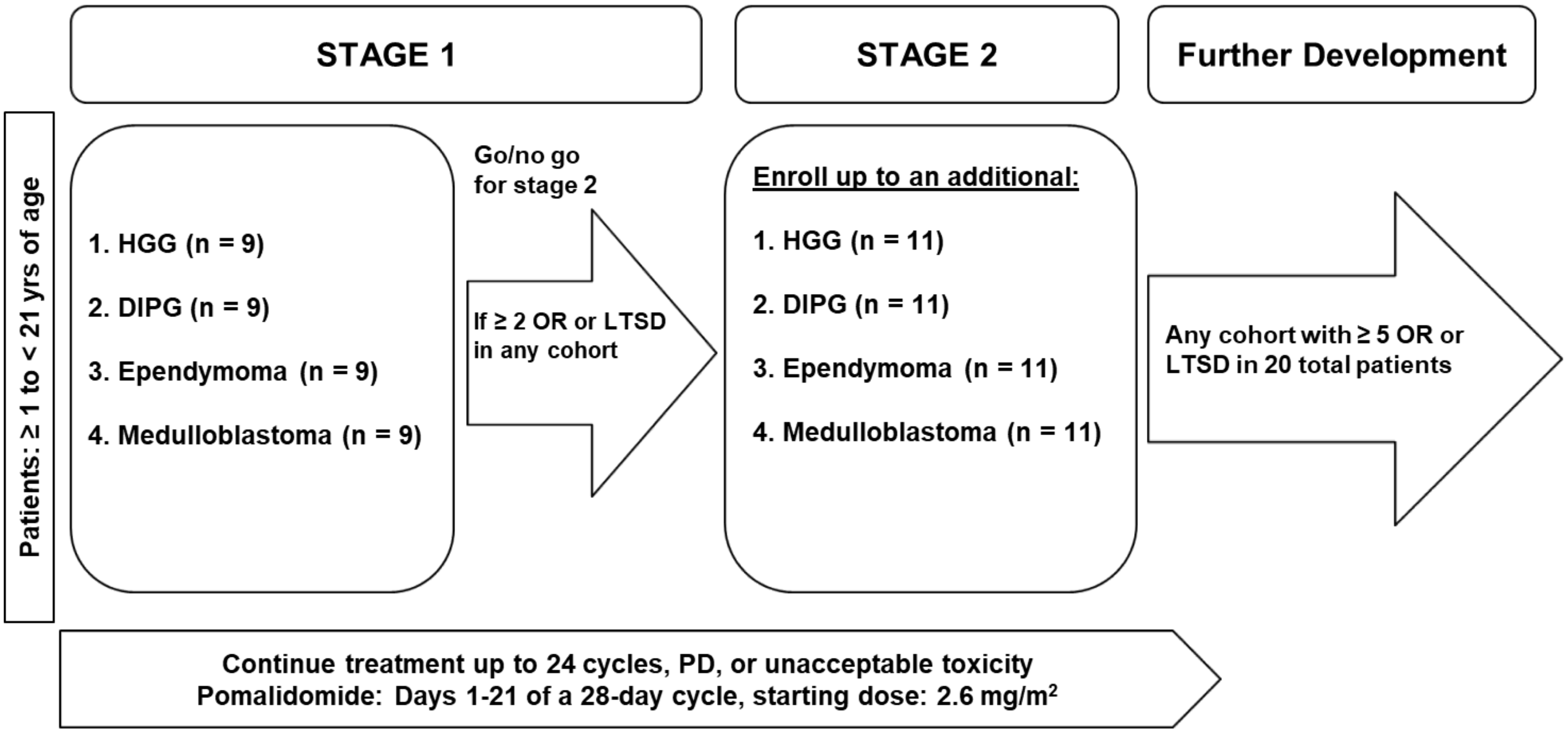

DIPG, diffuse intrinsic pontine glioma; HGG, high-grade glioma; LTSD, long-term stable disease (maintained for ≥ 6 cycles [≥ 3 cycles for DIPG]); OR, objective response (either complete or partial response).
